# An update on the tumor-suppressive functions of the RasGAP protein DAB2IP with focus on therapeutic implications

**DOI:** 10.1038/s41418-024-01332-3

**Published:** 2024-06-20

**Authors:** Rossella De Florian Fania, Arianna Bellazzo, Licio Collavin

**Affiliations:** 1 Department of Life Sciences, University of Trieste, Via L. Giorgieri 1, 34127 Trieste, Italy; 2 Unit of Immunopathology and Cancer Biomarkers, Centro di Riferimento Oncologico di Aviano (CRO), IRCCS, Via Franco Gallini, 2, 33081 Aviano, Italy

**Keywords:** Tumour-suppressor proteins, Cell biology

## Abstract

The dynamic crosstalk between tumor and stromal cells is a major determinant of cancer aggressiveness. The tumor-suppressor DAB2IP (Disabled homolog 2 interacting protein) plays an important role in this context, since it modulates cell responses to multiple extracellular inputs, including inflammatory cytokines and growth factors. DAB2IP is a RasGAP and negatively controls Ras-dependent mitogenic signals. In addition, it modulates other major oncogenic pathways, including TNFα/NF-κB, WNT/β-catenin, PI3K/AKT, and androgen receptor signaling. In line with its tumor-suppressive role, DAB2IP is frequently inactivated in cancer by transcriptional and post-transcriptional mechanisms, including promoter methylation, microRNA-mediated downregulation, and protein-protein interactions. Intriguingly, some observations suggest that downregulation of DAB2IP in cells of the tumor stroma could foster establishment of a pro-metastatic microenvironment. This review summarizes recent insights into the tumor-suppressive functions of DAB2IP and the consequences of its inactivation in cancer. In particular, we explore potential approaches aimed at reactivating DAB2IP, or augmenting its expression levels, as a novel strategy in cancer treatment. We suggest that reactivation or upregulation of DAB2IP would concurrently attenuate multiple oncogenic pathways in both cancer cells and the tumor microenvironment, with implications for improved treatment of a broad spectrum of tumors.

## Facts

- The tumor-suppressor DAB2IP is a RasGAP that also functions as a signaling modulator of several key oncogenic pathways.
- The loss of DAB2IP in cancer cells fosters tumor growth, metastasis, and chemo- and radioresistance.
- DAB2IP is rarely mutated in cancer but is frequently downregulated or inactivated by transcriptional and post-transcriptional mechanisms.
- Reactivation of DAB2IP function in cancer cells counteracts oncogenic phenotypes.

## Open questions

- Which is the specific contribution of DAB2IP inactivation in generating a pro-metastatic microenvironment?
- Is loss of DAB2IP in non-transformed stromal cells relevant for the growth and progression of tumors?
- Is the specific DAB2IP reactivation in cancer and stromal cells sufficient to inhibit the progression of cancers that bear different oncogenic mutations?
- Could pharmacological inhibition of the mechanisms of DAB2IP downregulation or inactivation be used to improve the efficacy of existing therapies?

## Introduction

Among the many genes whose dysregulation impacts cancer evolution and drug sensitivity, the tumor-suppressor DAB2IP (Disabled-2 Interacting Protein), also known as AIP (ASK1-interacting protein AIP1) is particularly interesting as it can negatively modulate several major oncogenic pathways. In fact, DAB2IP is a Ras GTPase-activating protein (GAP) and a cytoplasmic adapter for various signaling pathways. In particular, it modulates cell responses to growth factors and inflammatory cytokines, inhibiting oncogenic pathways such as NF-κB, WNT/β-catenin, PI3K/AKT, JAK/STAT, MAPK, and androgen receptor (AR) signaling. It also favors activation of the pro-apoptotic ASK1/JNK pathway, and contributes to genomic stability by modulating the function of PLK1 during mitosis [1–4]. DAB2IP is encoded by a complex gene with at least 4 different transcription start sites (TSS). Additionally, the last exon undergoes alternative splicing, resulting in two different C-terminal sequences (data from NCBI, Homo sapiens Annotation Release 150). As a consequence, different N- and C-terminal combinations encode several DAB2IP isoforms, whose specific functions in physiology and pathology remain largely unknown.

In multiple solid tumors, DAB2IP is clearly downregulated and its expression levels negatively correlate with tumor growth, angiogenesis, lymph node metastasis, advanced clinical stage, and resistance to therapies, establishing it function as a tumor suppressor [5–9]. Although its relevance in tumor initiation remains undefined, available evidences strongly indicate that loss of DAB2IP represents an advantage for cancer progression and metastasis [1, 2, 10, 11]. Notably, its mutation rate in cancers is relatively limited, and its loss-of-function is most frequently dependent on transcriptional silencing or post-transcriptional inactivation mechanisms; this makes DAB2IP a strong candidate for the development of therapeutics aimed to restore its tumor-suppressive function. Observations made in the last decade revealed novel regulatory mechanisms involved in modulating DAB2IP action, and further confirmed its role as a tumor suppressor and promising therapeutic target. Interestingly, some of these evidences suggest that DAB2IP may be an important player in the cancer-stroma crosstalk. Here, we review the most recent publications concerning the impact of DAB2IP loss-of-function in tumors, focusing on strategies that may be adopted to counteract its inhibition in cancer.

## DAB2IP loss of function promotes cancer growth and dissemination

DAB2IP mediates tumor suppression ensuring the inhibition of multiple oncogenic pathways, as it has been thoroughly reviewed previously [1, 2, 10, 11]. Recent studies showed that DAB2IP counteracts tumor growth and metastasis also cooperating with other tumor suppressors. For instance, in clear cell renal cell carcinoma (CCRC), depletion of DAB2IP was correlated to AKT-dependent inactivation of p27, with increased proliferation and tumor growth in mouse xenografts [12]. Intriguingly, DAB2IP was shown to mediate the stability of wild-type p53 in colon cancer cells by directly interacting with its negative regulator GRP75 [13]. Notably, forced expression of wild-type p53 impaired the growth of tumors subcutaneously transplanted into nude mice, but was ineffective if the tumors were depleted for DAB2IP. This suggests that DAB2IP may be necessary to promote the anti-cancer effects elicited by p53 ectopic expression. Accordingly, DAB2IP expression is positively associated with improved prognosis of colon cancer in patients with wild-type p53 tumors, in line with the notion that DAB2IP could support the tumor-suppressive action of p53 [13].

An analogous mechanism of action was reported linking DAB2IP loss-of-function with increased formation of invadopodia in mammary cells. Specifically, the competitive interaction of DAB2IP with the deubiquitinase USP10 was shown to be important to keep in check the levels of ALK kinase, thus limiting formation of actin-based invadopodia. Mammary epithelial cells depleted of DAB2IP reduce ALK turnover and, as a consequence, active ALK accumulates at the membrane periphery, promoting formation of invadopodia and invasive cell behavior, both in tissue culture and in nude mice xenografts [14].

By similar means, DAB2IP promotes the degradation of key oncogenic proteins. In various cell models, DAB2IP was shown to foster GSK3β activation, thus counteracting β-catenin nuclear accumulation [15]. In colorectal carcinoma DAB2IP cooperates with GSK3β and PP2A-B56α to dephosphorylate c-Myc and disrupt its stability [16]. DAB2IP also acts as a negative modulator of HSP90AA1 in colorectal cancer; through an unknown mechanism, DAB2IP inhibits HSP90AA1-mediated upregulation of SRP9, fostering activation of the pro-apoptotic JNK/ASK1 pathway and counteracting metastasis [17].

In prostate cancer, DAB2IP prevents EMT induced by IFN-γ treatment by dampening the JAK/STAT1 response and preventing upregulation of IFIT5, a gene involved in XRN1-mediated turnover of the anti-metastatic microRNA miR-363 [18].

In breast cancer, Cichowski and colleagues found that DAB2IP cooperates with another RasGAP family member, RASAL2, to limit metastasis in ER+ breast cancer. Intriguingly, they found that RASAL2 is frequently silenced together with DAB2IP in high-grade luminal B breast cancers [19]. In mouse xenografts, loss of either RASAL2 or DAB2IP alone was sufficient to promote tumor growth. In addition, the loss of both genes dramatically increased invasion and metastasis, suggesting a non-redundant action of the two proteins in suppressing the invasive phenotype [19]. More recently, the same group reported that in a fraction of colorectal cancers DAB2IP is mutated or silenced regardless of RAS or BRAF mutation, strongly indicating that DAB2IP loss-of-function may provide aggressive phenotypes independently of RAS hyperactivation [20].

A recent study involved DAB2IP in formation of the primary cilium, an organelle that functions as a sensor of extracellular physical and biochemical signals. In renal epithelial cells, DAB2IP participates to the stabilization of the primary cilium via direct interaction with kinesin family member 3A (KIF3A), a protein necessary for cilium formation [21]. Since the primary cilium regulates multiple signaling pathways including Hedgehog, WNT/β-catenin, G-protein-coupled receptors, TGF-β receptors, and various Tyrosine kinases, it is possible that DAB2IP loss-of-function may lead to hyperactivation of pro-tumorigenic pathways also by disrupting cilium functions.

A few studies linked DAB2IP inactivation to alterations in cell metabolism. DAB2IP expression negatively correlated with Type 2 diabetes in the adipose tissue of obese patients, and in vitro studies showed reduced glucose uptake in DAB2IP-depleted adipocytes [22]. Moreover, DAB2IP-depletion in HUVECs correlated with increased HIF-1α and VEGF, and promoted proliferation, motility, and tube formation under high-glucose conditions [23]. A recent study found that DAB2IP can modulate glucose metabolism during hypoxia by promoting degradation of HIF-1α; in breast cancer cells cultured in low oxygen, DAB2IP depletion increased glucose uptake, lactate production, and intracellular ATP levels, while DAB2IP overexpression had opposite effects [24].

**Fig. 1:**
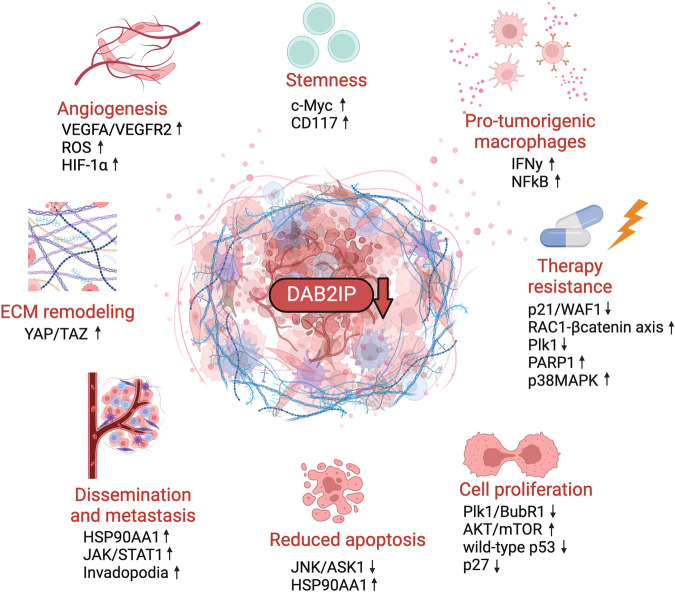
Summary of the oncogenic effects of DAB2IP downregulation in cancer. Inactivation or downregulation of DAB2IP in cancer cells and in stromal cells of the tumor microenvironment (TME) impacts multiple hallmarks of aggressiveness. DAB2IP loss in tumor cells favors the acquisition of stem cell-like properties, supports the activation and recruitment of pro-tumorigenic macrophages, increases resistance to chemo- and radio-therapy, promotes cell proliferation, dampens apoptosis, and contributes to metastatic dissemination. DAB2IP downregulation might also influence the extracellular matrix by activating YAP/TAZ signaling. Loss of DAB2IP in endothelial cells promotes neo-angiogenesis and favors metastasis. Some of the most recently described molecular events linked to DAB2IP inactivation are indicated, with arrows referring to upregulation or downregulation of corresponding genes or pathways. See text for details and references (created with BioRender.com).

Interestingly, evidences suggest that DAB2IP may respond to physical alterations of the tumor microenvironment (TME) and, consequently, modulate cell behavior upon mechanical stimuli. Zhang and colleagues reported that colon cancer cells grown in a soft fibrin matrix significantly reduce DAB2IP levels, and this correlates with Nanog upregulation and enhanced proliferation and self-renewal, both in vitro and in mouse xenografts [25]. Although the molecular mechanism was not explored, the data suggest that a soft extracellular environment induces DAB2IP downregulation and this facilitates acquisition of a cancer stem cell-like phenotype. In 2D cultures of epithelial mammary cells, we recently observed that DAB2IP protein levels increase with confluency and decrease when cells lose contact with adjacent cells, or are detached from the substrate. We found that DAB2IP knockdown in confluent cells stimulates morphological changes such as increased nuclear area and cell stiffness, along with YAP/TAZ nuclear localization and transactivation activity [26]. Although the molecular mechanism remains to be defined, these data suggest that DAB2IP loss-of-function could promote oncogenesis also by loosening control over YAP/TAZ in epithelial tissues. Phenotypes correlated to DAB2IP inactivation are summarized in Fig. 1.

In line with the effects of its depletion or inhibition, several studies over the past 20 years confirmed that DAB2IP overexpression dampens cancer cell growth, metastasis and stem cell phenotypes, in different tumor types (Table 1). Early studies in prostate cancer (PCa) cells showed that DAB2IP overexpression downregulates EMT markers and inhibits tumor growth and metastasis [27]. In recurrent prostate cancer, the expression of DAB2IP is inversely correlated with androgen receptor activation, and DAB2IP expression in PCa cells can suppress androgen-induced cell proliferation and gene activation [28]. More recently, it was reported that DAB2IP overexpression in PCa cells repressed transcription of the steroidogenic enzyme AKR1C3, reducing intra-tumoral testosterone levels under castrated condition [29].

**Table 1:** A selection of published evidence of the effects of DAB2IP overexpression in different tumor types. Molecular mechanisms are also summarized.

Tumor type	Mechanism	Biological effect	Ref.
Prostate cancer	Inhibition of Ras and NF-κB;	Reduced tumor growth in xenografts; reduced chemoresistance in vitro	[27]
	Suppression of Egr-1 and Clusterin expression;	Reduced chemoresistance in vitro	[5]
	Inhibition of c-kit dependent Myc and ZEB1 expression	Reduced stem-like behavior and sphere formation	[45]
Bladder cancer	Inhibition of STAT3 activation; suppression of Twist1 and P-glycoprotein expression	Reduced resistance to pirarubicin in vitro	[39]
Pancreatic cancer	Decreasing levels of p-AKT and p-ERK, and inhibition of Ras activity; increasing levels of p-JNK and active caspase 3	Inhibition of cell proliferation, invasiveness and migration; increased sensitivity to cetuximab in vitro	[40]
Gastric cancer	Inhibition of AKT and ERK activation	Impaired cell proliferation, and increased sensitivity to cisplatin in vitro	[41]
Breast cancer	Inhibition of insulin-induced AKT activation	Reduced insulin-induced proliferation in vitro	[72]
	Blocking RAC1-dependent β-catenin nuclear localization	Reduced spheroid growth and increased sensitivity to Docetaxel	[36]
Nasopharyngeal carcinoma (NPC)	Inhibition of the PI3K/AKT pathway	Reduced subcutaneous growth and lung metastasis in nude mice	[30]
Osteosarcoma	Inhibition of AKT and ERK activation	Reduced proliferation, migration and invasion in vitro	[31]
Esophageal squamous cell (ESCC) carcinoma	Enhanced IR-induced activation of the ASK1/JNK pathway	Increased apoptosis after cisplatin treatment and ionizing radiation (IR) in vitro; reduced tumor size after IR in vivo	[44]
Colorectal cancer	Inhibition of AKT and ERK activation; regulation of HSP90AA1/SRP9/ASK1/JNK signaling axis	Decreased cell growth and migration in vivo and increased sensitivity to chemotherapeutic drugs in vitro	[42]
	Inducing c-Myc degradation via GSK3β- mediated phosphorylation	Impaired growth and self-renewal of tumor-repopulating cells	[16]
	Inhibition of Ras-ERK and NF-κB	Reduced tumor formation and progression in vivo	[20]
Glioblastoma	Suppression of ATG9B expression by blocking the Wnt/β-catenin pathway	Reduced autophagy-mediated chemoresistance to temozolomide in vitro	[43]

Similarly, DAB2IP overexpression impaired proliferation, migration, and invasion of nasopharyngeal carcinoma (NPC) and osteosarcoma cells in vitro, and prevented lung metastasis of NPC injected in nude mice [30, 31]. Notably, in vivo, DAB2IP-mediated inhibition of cancer growth and metastasis can be uncoupled. In fact, a GAP-deficient mutant DAB2IP (R289L) was unable to suppress tumor growth, but significantly reduced metastasis, indicating that the RasGAP activity is required to inhibit tumor growth while other DAB2IP functions are involved in preventing tumor dissemination [27]. This dual action was confirmed recently in a colorectal cancer model where DAB2IP reconstitution inhibited tumor formation and progression in vivo; in this context, both the RasGAP domain and the period-like domain are necessary to suppress tumor development, in particular by preventing NF-κB signaling and the recruitment of pro-tumorigenic macrophages [20].

## DAB2IP loss promotes resistance to chemo- and radio-therapies

More than 10 years ago, Wu et al. observed that castration-resistant prostate cancers (CRPC) displayed lower levels of DAB2IP compared with androgen sensitive cancers, providing the first evidence that correlated its expression with resistance to therapy [5]. Zhou et al. further linked DAB2IP loss to castration resistance by showing that DAB2IP depletion enhanced STAT3 signaling and apoptosis resistance in androgen-deprived PCa cells [32]. In the following years additional evidences confirmed that DAB2IP loss contributes to resistance to chemo- and radio-therapy of multiple different cancers (Fig. 1). For instance, DAB2IP modulates resistance to mTOR inhibitors in advanced renal cell carcinoma; reduced DAB2IP expression caused by epigenetic silencing by EZH2 results in hyper-activation of extracellular signal-regulated kinase/RSK1 and the PI3K/mTOR pathway, which induces the expression of HIF-2α and the consequent inhibition of p21/WAF1 expression, associated with resistance to mTOR inhibitors [33, 34].

Similarly, DAB2IP loss contributes to docetaxel (DOC) resistance in triple-negative breast cancer (TNBC). Mechanistically, DAB2IP interacts with RAC1 and impedes RAC1-mediated β-catenin nuclear translocation, so that DAB2IP loss promotes the induction of cancer stem cell phenotypes and DOC resistance [35, 36].

Regarding radiotherapy, DAB2IP loss promotes bladder cancer cells resistance to ionizing radiation (IR) by increasing the expression of ataxia‐telangiectasia mutated (ATM) and the activation of NF-κB pathway and p38MAPK. The loss of ATM is sufficient to restore the sensitivity of DAB2IP-silenced cancer cells to radiotherapy by delaying double‐strand break (DSB) repair kinetics and counteracting the activation of NF-κB and p38MAPK [37].

More recently, Yun et al. provided a mechanism for the effects of DAB2IP loss in promoting IR resistance in renal cell carcinoma (RCC). Immunoprecipitation-mass-spectrometry analysis revealed that DAB2IP forms a complex with PARP-1 and E3 ligases, promoting proteasome-mediated PARP-1 degradation. Increased PARP-1 function is associated with IR resistance in RCC cells, given its role in resolving DNA double-strand breaks. Accordingly, PARP-1 inhibitors enhanced the IR response of RCC xenografts and patient-derived xenografts (PDX), and this effect was stronger in tumors expressing lower levels of DAB2IP [38].

Not surprisingly, the reconstitution of DAB2IP can increase the sensitivity of cancer cells to chemotherapy (Table 1). For instance, DAB2IP overexpression restored drug sensitivity in prostate cancer cells, by reducing the levels of the anti-apoptotic factor Clusterin [5]. Similarly, expression of DAB2IP increased the sensitivity of PCa cells to microtubule-stabilizing drugs (paclitaxel and docetaxel) and the PLK1 inhibitor BI2536 [3, 4]. In bladder cancer, DAB2IP overexpression was reported to reduce metastasis and chemoresistance in vitro by inhibiting STAT3 activation [39]. In pancreatic cancer, overexpressed DAB2IP increased sensitivity to cetuximab of cancer cells in vitro, and tumor growth in vivo in mouse xenografts [40]. In gastric cancer, DAB2IP upregulation impaired cell proliferation and sensitized cancer cells to cisplatin, while DAB2IP depletion had opposite effects [41]. Almost identical observations were reported in colorectal cancer, where the expression of DAB2IP sensitized cancer cells to cisplatin, oxaliplatin, and doxorubicin, and inhibited tumor growth in mouse xenografts, with reduced AKT and ERK activation [42]. In glioblastoma, overexpression of DAB2IP could lower the intrinsic resistance of these cancer cells to temozolomide [43]. In esophageal squamous cell carcinoma (ESCC) cells, expression of DAB2IP was shown to reduce resistance to ionizing radiation; in vitro, DAB2IP overexpressing cells displayed higher apoptosis after cisplatin treatment and IR exposure. In nude mice, DAB2IP expression did not affect the growth rate of ESCC xenografts, but after IR treatment DAB2IP-expressing tumors were significantly reduced compared to controls [44].

Finally, DAB2IP has been reported to inhibit the stem cell properties of cancer cells, with implications for progression and chemoresistance. In PCa cells, DAB2IP inhibited expression of stem cell factor receptor c-kit (or CD117) reducing the capability to generate tumor spheres in vitro [45]. In colon carcinoma cells, reconstitution of DAB2IP limited the number of cancer stem cells (CSC)-like tumor-repopulating cells by impairing c-Myc expression [16], while in triple-negative breast cancer, DAB2IP-overexpression inhibited spheroid formation from single cells and reduced chemoresistance to docetaxel [36].

## DAB2IP loss supports a pro-metastatic tumor microenvironment

Interestingly, it appears that DAB2IP inactivation generates a pro-tumoral inflammatory microenvironment that affects both cancer progression and response to therapy (Fig. 1). Ten years ago, our group reported a pro-invasive gene expression response to TNFα caused by DAB2IP inactivation in breast cancer cells expressing mutant p53 [46]. We later confirmed that DAB2IP loss-of-function amplifies NF-κB activation and contributes to a pro-inflammatory gene expression pattern in breast and prostate cancer cells [47]. More recently, Cichowski and colleagues elegantly showed that loss of DAB2IP triggers the production of inflammatory mediators and the onset of a pro-tumorigenic immune microenvironment in KRAS-mutant colon cancer [20]. When DAB2IP-depleted cancer cells were xenografted in nude mice, the resulting tumors recruited abundant macrophages; strikingly, elimination of macrophages by treatment with liposomal clodronate suppressed tumor formation, establishing a key role for the infiltrate in this model [20]. Although experiments were done in nude mice, they indicate that DAB2IP status can potentially influence the immune landscape of tumors, with implications for progression and therapy response.

According to public gene-expression databases, DAB2IP is not transcribed in blood and immune cells, being only detected in thymocytes (Gene Expression Atlas https://www.ebi.ac.uk/gxa/; GTExPortal https://www.gtexportal.org/). However, it cannot be excluded that molecules secreted by cancer cells might affect its expression in tumor-infiltrating immune cells. The effects of DAB2IP expression in immune cells are essentially unexplored, except for one study reporting that DAB2IP is downregulated during positive selection of immature thymocytes in mice, and deletion of the CCR4-NOT complex abolishes such downregulation, correlating with increased cell death [48]. A better understanding of the role of DAB2IP in immune cells may offer useful insights on its potential in modulating the tumor immune environment.

Notably, the altered secretome of DAB2IP-depleted cancer cells also affects tumor vascularization, with implications for aggressiveness. In fact, a recent study showed that xenografted tumors developed from DAB2IP-knockdown cancer cells have higher micro-vascular density and increased radioresistance [49].

Less is known about the potential role of DAB2IP in non-transformed cells of the tumor stroma, but there is clear evidence that its loss in endothelial cells (EC) promotes neo-vascularization and angiogenesis [50, 51]. Perhaps the most convincing study was published by Wang Min and colleagues, who found that conditional DAB2IP depletion in ECs is sufficient to boost tumor growth and metastasis in mouse xenograft models of melanoma and breast cancer. They found that loss of DAB2IP in ECs amplifies VEGFR2 signaling and the secretion of molecules involved in EMT and angiogenesis, and this increases tumor neovascularization and generates a pre-metastatic microenvironment [52]. The same authors observed that EC-specific DAB2IP knockdown amplifies ROS production and vascular inflammation, with consequent increase of atherosclerosis [51]; this may also indirectly contribute to establish a pro-metastatic microenvironment.

**Fig. 2:**
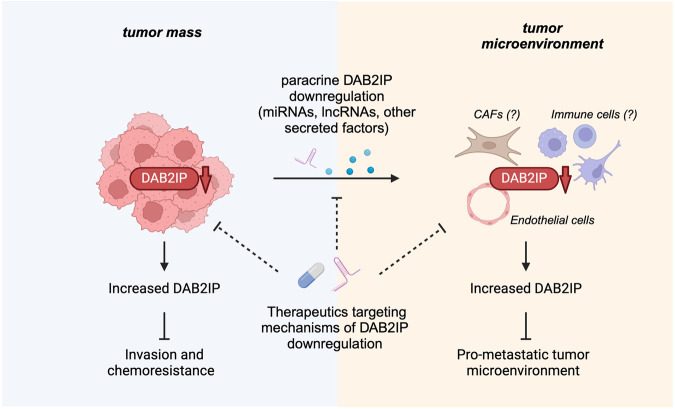
Potential impact of DAB2IP reactivation in cancer cells and in the tumor microenvironment. Cell autonomous and cell non-autonomous downregulation of DAB2IP affects the behavior of cancer and stromal cells, modulating the immune infiltrate, promoting neo-angiogenesis, and potentially reshaping the tumor microenvironment. In this framework, therapeutics that counteract cellular and/or extracellular mechanisms of DAB2IP inactivation, or that simply increase DAB2IP expression levels, could act both on cancer cells and stromal cells to promote tumor suppression and improve response to therapies. DAB2IP reactivation in cancer cells would limit their intrinsic invasiveness and chemoresistance. DAB2IP upregulation in endothelial cells would limit neoangiogenesis and curb tumor dissemination. We further speculate that promoting DAB2IP activity in other cell types of the tumor stroma could also contribute to prevent establishment of a pro-metastatic TME (created with BioRender.com).

Together, these observations indicate that DAB2IP in endothelial cells is indeed important to limit tumor dissemination, thus posing the interesting question of whether cancer cells might actively inhibit DAB2IP in neighboring stromal cells - endothelial cells but perhaps also fibroblasts and other cell types - to establish a pro-metastatic environment (Fig. 2).

As a partial answer to this question, we and others found that treatment with conditioned medium from breast and prostate cancer cells induces DAB2IP downregulation in receiving non-transformed cells, stimulating proliferation, migration, and tube-forming ability of endothelial cells [47, 49, 53]. We found that miR-149-3p is secreted by cancer cells and contributes to DAB2IP downregulation in non-transformed cells [47]. Similarly, Zhu et al. found that clear cell renal carcinoma (CCRC) cells secrete the long non-coding RNA (lncRNA) DMDRMR, which acts as a sponge for miR-378a-5p, and indirectly causes DAB2IP downregulation in receiving cells; this correlates with enhanced VEGFA/VEGFR2 signaling and increased tube formation by HUVECs [53]. Together, these data support the hypothesis that cancer cells can induce a pro-metastatic TME by paracrine downregulation of DAB2IP in stromal cells. Importantly, they also imply that pharmacological targeting of such cell non-autonomous mechanisms could counteract the pro-metastatic effects caused by the loss of DAB2IP function in cells of the TME (Fig. 2).

## Blocking mechanisms of DAB2IP inhibition may be sufficient to revert aggressive phenotypes in various tumors

**Fig. 3:**
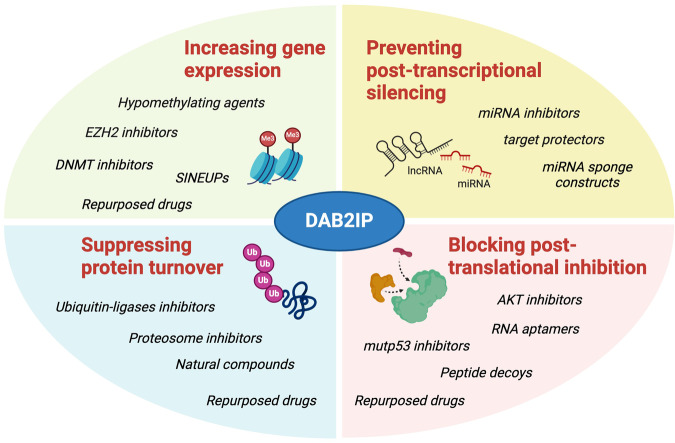
Schematic summary of approaches that may be implemented to counteract DAB2IP inactivation or increase DAB2IP protein levels in cancers. DAB2IP is most frequently inactivated by epigenetic silencing, post-transcriptional downregulation by micro RNAs, post-translational inhibition, and ubiquitin-mediated degradation. These mechanisms can be potentially targeted by developing novel therapeutics or by repurposing already existing drugs. For instance, hypomethylating agents or histone methyltransferase inhibitors may be used in tumors where DAB2IP is epigenetically silenced. Also, ncRNAs customized to enhance mRNAs translation (e.g. SINEUPs) may be employed to increase DAB2IP protein levels. Post-transcriptional silencing may be prevented by blocking the action of DAB2IP-targeting miRNAs with antisense inhibitors, sponge constructs, or target-protector oligonucleotides. DAB2IP post-translational inactivation may be counteracted by preventing the formation of protein complexes (e.g. with mutp53) using peptide or RNA aptamers, or by interfering with inhibitory phosphorylation (e.g. by AKT) using specific kinase inhibitors. Finally, DAB2IP degradation may be controlled using proteasome inhibitors or drugs targeting specific ubiquitin-ligases (created with BioRender.com).

Most DAB2IP reconstitution studies involved ectopic expression at non-physiological levels of a single isoform, and for this reason, they must be taken with caution. However, there are a few studies in which authors have been able to restore physiological levels, or functions, of DAB2IP in cancer cells by interfering with known mechanisms of its inactivation. This seems to be sufficient to induce the anti-tumoral effects observed with DAB2IP ectopic expression, thus suggesting a potential therapeutic strategy (Fig. 3).

### Alleviating transcriptional repression

In many tumors, DAB2IP transcription is reduced by DNA methylation at various CpG islands (reviewed in [1, 2, 10, 54]). Recent observations confirmed that DAB2IP is downregulated by promoter methylation in luminal B breast cancers [19], and that CpG methylation is inversely correlated with overall survival of patients with renal cell carcinoma, suggesting DAB2IP methylation as a prognostic indicator for this type of cancer [55]. DAB2IP transcription is also regulated by histone methylation, mostly by the Polycomb-repressive complex 2 (PCR-2)-Enhancer of Zeste homolog 2 (EZH2) [27, 56]. Therefore, targeting the mechanisms responsible of DAB2IP epigenetic silencing may be a valid strategy to re-establish its tumor-suppressive function in cancer (Fig. 3).

De-methylating agents (e.g. azacitidine, decitabine) are currently used in monotherapy as well as in combination with traditional chemotherapeutics to treat different types of malignancies [57]. Targeting the epigenetic regulator EZH2 with the inhibitor GSK-126 induced the regression of CRPC in vivo, providing clear evidence that inhibiting transcriptional repressive signals may be a valid strategy to fight aggressive tumors [58]. In this regard, there are evidences that de-methylating agents can restore DAB2IP expression and attenuate chemoresistance. For instance, inhibition of DAB2IP promoter methylation via knockdown of the DNA methyltransferase 3A (DNMT3A) reduced proliferation and survival of colorectal cancer cells by counteracting the activation of the MEK/ERK signaling pathway, possibly as a consequence of increased DAB2IP levels [59]. Xiong et al. reported the additive action of decitabine with docetaxel in MDA-MB-231 xenografts, linking this effect to DAB2IP-mediated sequestration of RAC1 [36]. In another study, treatment with the EZH2 inhibitor GSK-126 in combination with the RAC1 inhibitor NSC23766, eliminated ovarian cancer stem cells in vitro and increased platinum sensitivity in vivo, probably by synergically reinforcing the inhibitory effect on WNT signaling consequent to DAB2IP restoration [9] (Table 2).

**Table 2:** A survey of pharmacological approaches that could be used to restore DAB2IP function in cancer, or may be employed to specifically target DAB2IP-deficient tumors.

	Treatment	Examples	Effects	Ref.
Approaches to restore DAB2IP function or increase DAB2IP levels	De-methylating agents	Decitabine	DAB2IP upregulation. Synergic action with docetaxel in reducing growth of breast cancer xenografts	[36]
		EZH2 inhibitors (GSK-126)	DAB2IP upregulation. Regression of castration-resistant prostate cancer in vivo; elimination of ovarian cancer stem cells in vitro and increased platinum sensitivity in vivo in combination with the RAC1 inhibitor NSC23766	[9, 58]
		DNA methyltransferase 3 A inhibitors	DAB2IP upregulation. Reduction of proliferation and survival of colorectal cancer cells	[59]
	RNA-based therapeutics	miR-1266 inhibitor	DAB2IP upregulation. Reduction of cell viability, proliferation, migration and invasion of cervical cancer cells	[61]
		Anti-miR-182	DAB2IP upregulation. Reduction of colorectal cancer growth in vivo	[62]
		Anti-miR-1307-3p	DAB2IP upregulation. Repression of hepatocellular carcinoma proliferation and invasion in vitro and tumor growth in vivo	[63]
		miR-149-3p sponge	DAB2IP upregulation. Reduction of growth of prostate cancer xenografts	[47]
		circRNA-008913	DAB2IP upregulation. Reduction of growth and tumor formation of arsenite-transformed keratinocytes	[64]
		circRNA-5692	DAB2IP upregulation. Reduction of growth and tumor formation of hepatocellular carcinoma cells	[65]
	Mutant p53 inhibitors	DAB2IP-mutp53 protein decoy	Less mutant p53 binding to DAB2IP. Reduction of metastasis of breast cancer cells xenografts; repression of insulin-induced proliferation and invasion of breast cancer cells	[46, 72]
		HSP90 inhibitors, Atorvastatin, Trisenox, Vorinostat	Reduction of mutant p53 levels. Unkown effects on DAB2IP.	[74–76]
	AKT inhibitors	Capivasertib, Ipatasertib MK-2206	Predicted to reduce DAB2IP inhibitory phosphorylation	[69]
	Ubiquitin-ligase and proteasome inhibitors	Ubiquitin-ligase inhibitors	Predicted to limit DAB2IP degradation. Deletion of SMURF1 reduced proliferation, invasion and EMT in ovarian cancer cells	[80]
		Proteasome inhibitors (bortezomib, carfilzomib, ixazomib, marizomib, oprozomib, flavonoids)	Predicted to limit DAB2IP degradation	[81, 82]
Approaches to target DAB2IP-deficient tumors	Synthetic lethality-based strategies	PARP inhibitors (Olaparib)	Resensitization of DAB2IP-deficient renal cell carcinomas to IR	[38]
		Microtubule inhibitors (epothilone B)	Resensitization of DAB2IP-deficient prostate cancer cells to IR	[85]
		Cytolethal distending toxin (CDT)	Resensitization of DAB2IP-deficient prostate cancer cells to IR	[84]
		ATM inhibitors (KU55933)	Resensitization of DAB2IP-deficient bladder cancer cells to IR	[83]
		AR inhibitors (enzalutamide, abiraterone)	Potential efficacy in DAB2IP-deficient castrate-resistant prostate cancers	[70, 71]

Epigenetic drugs are inevitably non-specific, but alternative approaches can be developed to increase expression of a tumor-suppressor gene such as DAB2IP that is silenced but not deleted in cancer. One example may be the use of SINEUPs, a recently discovered class of non-coding RNAs (ncRNAs) that pair with complementary target messenger RNAs and recruit the ribosome to increase protein synthesis [60].

### Blocking post-transcriptional downregulation

DAB2IP has a long 3’UTR sequence and is targeted by several intracellular and secreted microRNAs (miRNAs) [2, 10, 47]. There are various evidence that blocking inhibitory miRNAs can increase endogenous DAB2IP protein levels and restore its onco-suppressive functions. For instance, miR-1266 targets DAB2IP and transfection of a miR-1266 inhibitor reduced cell viability, proliferation, migration and invasion in HeLa and SiHa cells by increasing DAB2IP expression [61]. Similarly, miR-182 targets DAB2IP and expression of an anti-miR-182 inhibited colorectal cancer growth in vivo by upregulating DAB2IP [62]. In hepatocellular carcinoma cells (HCC), miR-1307-3p increased proliferation and invasion under hypoxia conditions by downregulating DAB2IP to activate AKT/mTOR and increase HIF-1α expression. Transfection of an anti-miR-1307-3p repressed HCC proliferation and invasion in vitro and tumor growth in vivo; importantly the effect was abrogated in DAB2IP knockdown cells, confirming its dependency on DAB2IP upregulation [63]. Similarly, a moderate increase of DAB2IP protein, obtained by inhibition of miR-149-3p, was sufficient to suppress oncogenic phenotypes of PCa cells in vitro. Likewise, expression of a sponge construct targeting miR-149-3p increased endogenous DAB2IP levels and reduced growth of PC3 xenografts, both in zebrafish embryos and in NOD/SCID mice [47].

Coherent results were reported in studies on circRNA-008913 and circRNA-5692, two circular RNAs that act as sponges for DAB2IP-targeting miR-889 and miR-328-5p, respectively. Stable expression of these circRNAs reduced the capacity of CSC-like keratinocytes and hepatocellular carcinoma cells to form colonies, invade, migrate, and generate tumors in nude mice; this correlated with upregulation of endogenous DAB2IP [64, 65] (Table 2).

Together these evidences support the hypothesis that interfering with miRNA-mediated inhibition may restore the tumor-suppressive function of DAB2IP and revert aggressive phenotypes caused by the loss of its function both in tumor and in nearby stromal cells. RNA-based therapies are well developed and anti-miRNAs are already in clinical trials. Various molecules can be used to inhibit activity of specific miRNAs, such as chemically modified antisense oligonucleotides (ASO) that bind mature miRNAs, or locked nucleic acids (LNA) that mask miRNA seed sequences on the target mRNA and act as target protectors [66] (Fig. 3).

The major limit to the clinical use of RNA-based approaches is their delivery to the tumor cells. Intense research is ongoing to develop lipid and non-lipid nanocarriers for RNA delivery, and even small molecule-based therapies to target RNA are currently under investigation (reviewed in [66]). The fact that molecules secreted by cancer cells downregulate DAB2IP in stromal cells, and that DAB2IP likely plays a tumor-suppressive role in the TME (discussed above), implicate that RNA-based therapeutics aimed at increasing DAB2IP levels may not need to reach the core of solid tumors to be clinically effective. Further research will eventually clarify whether DAB2IP itself and/or some of its negative regulators are indeed usable targets for the development of this kind of drugs.

### Targeting post-translational inhibition

There are various evidences of DAB2IP inactivation by post-translational modifications (PTMs) or protein-protein interactions (PPIs) that could be targeted to restore its function.

DAB2IP is phosphorylated by AKT1 within its proline-rich domain, and this modification blocks efficient binding to its functional partners RAS and TRAF2, and stimulates its degradation [67, 68]. Preventing DAB2IP phosphorylation by AKT may thus be a strategy to restore its tumor-suppressive function. A number of molecules targeting AKT have been developed to block its pro-oncogenic functions, and some inhibitors are being tested in phase III clinical trials [69]. In this regard, combination of AKT inhibitors with traditional chemotherapeutics apparently improved their anti-tumor efficacy in metastatic castrate-resistance prostate cancer patients [70, 71]. It would be worth investigating if the anti-cancer effects of AKT inhibition in certain tumors are mediated, at least in part, by restoration of DAB2IP functions.

Also, DAB2IP can be bound and functionally inhibited by mutant p53 in the cytoplasm of cancer cells. This inhibitory binding reprograms the way cancer cells respond to inflammatory cytokines and growth factors, enhancing tumor cells’ proliferation and invasiveness [46, 72]. Ectopic expression of a decoy protein capable of interfering with DAB2IP-mutp53 interaction reduced tumor growth and metastasis of breast cancer cells in a mouse xenograft model [46]. Similarly, such a decoy abolished insulin-induced proliferation and invasion in cultured breast cancer cells bearing mutp53 [72]. These results provide proof of concept that preventing mutp53-DAB2IP interaction can have tumor-suppressive effects. In recent years, PPIs modulators have entered clinical studies and some of them have already been approved for cancer treatment; they include small molecules, peptides, antibodies, and RNA aptamers [73]. Designing molecules able to inhibit the DAB2IP-mutp53 interaction may be a strategy to restore the tumor-suppressive function of DAB2IP in cancers that bear p53 missense mutations (Fig. 3).

Alternatively, pharmacological approaches to destabilize mutp53 have been developed and are effective in vitro and in preclinical models, and some of them have already reached clinical stage [74–76]. It would be interesting to test whether the tumor-suppressive effects of mutp53 inhibitors are at least in part mediated by DAB2IP, and/or whether DAB2IP stabilizing agents could increase the anti-tumoral effects of mutp53-targeting drugs (Table 2).

### Blocking protein degradation

Despite DAB2IP being a rather stable protein, various ubiquitin-ligases have been reported to influence its turnover in cancer cells. Tsai et al. reported that the E3 ubiquitin-ligase S-phase kinase-associated protein-2 (Skp2) can promote DAB2IP N-terminal ubiquitination and proteasome-mediated degradation in normal and cancer prostate cells [77]. Dai et al. reported that DAB2IP can be degraded by the ubiquitin-ligase complex SCF/Fbw7 after phosphorylation by AKT in prostate and colon cancer cells [68]. Finally, the E3 ubiquitin-ligase SMURF1 (SMAD ubiquitylation regulatory factor-1), was reported to induce DAB2IP ubiquitination and elimination, promoting proliferation and invasion of ovarian and gastric cancer cells [78, 79]. Notably, deletion of SMURF1 in ovarian cancer cells increased DAB2IP levels and reduced proliferation, invasion and EMT; depletion of DAB2IP restored proliferation and EMT in SMURF1-knockout cells, suggesting that SMURF1 is required to maintain low levels of DAB2IP in ovarian cancer [80]. This last evidence suggests that targeting the ubiquitin-ligases responsible of DAB2IP ubiquitination may be a therapeutic strategy to reduce cancer aggressiveness by increasing DAB2IP levels.

Approaches that target the ubiquitin-proteasome system show efficacy in the treatment of many cancers, mostly by triggering cell death. The proteasome inhibitors bortezomib, carfilzomib, and ixazomib have been approved by the Food and Drug Administration for treating multiple myeloma and are currently in clinical trials for additional uses [81]. Not only, considerable research has been conducted in the areas of natural compounds and drug repositioning toward the aim of discovering effective and low toxicity proteasome-inhibitory drugs [82]. Similar to DNA methylation inhibitors, proteasome inhibitors lack specificity and can have unpredictable effects. However, it is possible that approved proteasome drugs may be effective in tumors where DAB2IP is destabilized by the ubiquitin-proteasome system, selectively increasing its levels (Fig. 3) (Table 2).

## Specifically targeting DAB2IP-deficient tumors may offer additional therapeutic opportunities

As an alternative to DAB2IP upregulation or reactivation, it may be possible to devise therapeutic strategies aimed to specifically target DAB2IP-deficient tumors (Table 2).

In early studies with prostate and bladder cancer cell lines, DAB2IP knockdown increased resistance to ionizing radiation but rendered them sensitive to combined treatment with genotoxic drugs such as the bacterial cytolethal distending toxin (CdtB) or an ATM inhibitor [83, 84]. Similarly, renal cell carcinoma cells with low DAB2IP levels are resistant to IR but specifically sensitive to treatment with radiotherapy plus PARP inhibitors [38].

Saha and colleagues found that DAB2IP plays a role in the spindle assembly checkpoint (SAC) by contributing to the activation of PLK1 during mitosis; accordingly, DAB2IP loss impairs checkpoint functions, promoting tumor aneuploidy and related aggressiveness [3, 4]. Theoretically, this can be exploited for targeted therapy of DAB2IP-deficient tumors that, given a poorly functional SAC, may be more sensitive to mitotic drugs. Indeed, radioresistant DAB2IP-knockdown PC3 cells were sensitive to IR treatment combined with the microtubule inhibitory drug Epothilone B [85].

These evidences confirm that specific treatments may be employed to increase the response to radiotherapy of aggressive DAB2IP-deficient tumors. Additional specific dependencies may be targetable in these tumors; for instance, since DAB2IP levels show strong inverse correlation with AR activation in CRPC [28, 29], DAB2IP-deficient prostate cancers may be particularly sensitive to AR inhibitors.

In this perspective, powerful insights may come from genomic and transcriptomic analysis of publicly available cancer datasets, such as The Cancer Genome Atlas (TCGA), that could reveal pathways or biological processes preferentially activated in DAB2IP-deficient cancers, possibly uncovering novel targetable mechanisms to induce synthetic lethality in these tumors.

## Conclusions

Multiple mechanisms of DAB2IP inactivation have been observed in cancer, suggesting that tumor cells take advantage from the inhibition of such a protein that negatively modulates several oncogenic signaling pathways. As we have summarized here, several independent studies confirmed that ectopic overexpression of DAB2IP is sufficient to counteract oncogenic phenotypes in different cancer models (Table 1). We also reviewed evidences that treatments that restore endogenous DAB2IP levels or functions in cancer cells inhibit oncogenic features associated to its loss of activity (Table 2). Therefore, the pharmacological reactivation of DAB2IP might be an innovative and feasible strategy to reduce tumor growth and dissemination, and improve the clinical response to primary chemotherapy treatments.

It is worth recalling that DAB2IP is quite unique as a tumor suppressor, since it modulates multiple key pathways aberrantly activated in many cancers [1, 2, 10, 11]. Upregulating DAB2IP levels may therefore be effective in tumors driven by various different oncogenic mutations, including some against which specific treatments are not yet available, or are inefficient.

Another interesting point is that a significant tumor-suppressive effect could be obtained by increasing DAB2IP levels not only in cancer cells but also in non-transformed cells of the tumor microenvironment, in particular endothelial cells (Fig. 2). It is reasonable to assume that stromal cells can be reached by small molecules or RNA-based drugs much more efficiently than cancer cells in the core of a solid tumor, thus allowing to overcome some limits of drug delivery.

Developing tools to achieve the reactivation of DAB2IP might be reasonably feasible. In addition to epigenetic drugs and proteasome inhibitors, an effective approach would employ molecules that counteract DAB2IP-targeting ncRNAs. Similarly, non-coding RNAs that positively affect DAB2IP levels could be discovered and developed as drugs. Aptamers preventing formation of inhibitory complexes may also be developed. Finally, there may be drugs already approved for clinical use that directly or indirectly affect DAB2IP levels by targeting the mechanisms controlling its synthesis or turnover, and may be rapidly repurposed to this scope.

The perspective of pharmacological DAB2IP reactivation may be complicated by two issues. The first is the existence of multiple DAB2IP isoforms. A recent study reported the first evidence of isoform-specific activity: in mouse models of vascular disease, a shorter DAB2IP, lacking the N-terminal PH domain, enhanced TNFα-induced ROS generation and neointima formation, but opposite effects were observed with the full-length [86]. Specific isoforms could be differentially affected by therapeutics, with variable outcomes. The second is that, although infrequent, deleterious mutations in DAB2IP are found in tumors, in particular colorectal and breast cancers [19, 20]. In those cases, treatments aimed at increasing the endogenous protein levels would be ineffective and a genetic analysis of the tumor would be necessary to predict whether or not it may benefit from this approach.

Despite these potential pitfalls, pharmacological reactivation or upregulation of a single protein such as DAB2IP that negatively modulates multiple oncogenic pathways, could significantly improve treatment of many cancers with minimal side effects. Clearly it is a difficult challenge, but an in-depth understanding of the molecular mechanisms involved in DAB2IP inactivation may enable the development of ad hoc strategies to manipulate its expression and function, that could have a relevant impact for cancer therapy.
